# Wearable Sensors for COVID-19: A Call to Action to Harness Our Digital Infrastructure for Remote Patient Monitoring and Virtual Assessments

**DOI:** 10.3389/fdgth.2020.00008

**Published:** 2020-06-23

**Authors:** Dhruv R. Seshadri, Evan V. Davies, Ethan R. Harlow, Jeffrey J. Hsu, Shanina C. Knighton, Timothy A. Walker, James E. Voos, Colin K. Drummond

**Affiliations:** ^1^Department of Biomedical Engineering, Case Western Reserve University, Cleveland, OH, United States; ^2^Department of Electrical Engineering, Case Western Reserve University, Cleveland, OH, United States; ^3^Department of Orthopaedics, University Hospitals of Cleveland Medical Center, Cleveland, OH, United States; ^4^Division of Cardiology, Department of Medicine, David Geffen School of Medicine at UCLA, University of California, Los Angeles, Los Angeles, CA, United States; ^5^Frances Payne Bolton School of Nursing, Case Western Reserve University, Cleveland, OH, United States

**Keywords:** wearable sensors, COVID-19, pandemic, predictive analytics, remote patient monitoring

## Abstract

The COVID-19 pandemic has brought into sharp focus the need to harness and leverage our digital infrastructure for remote patient monitoring. As current viral tests and vaccines are slow to emerge, we see a need for more robust disease detection and monitoring of individual and population health, which could be aided by wearable sensors. While the utility of this technology has been used to correlate physiological metrics to daily living and human performance, the translation of such technology toward predicting the incidence of COVID-19 remains a necessity. When used in conjunction with predictive platforms, users of wearable devices could be alerted when changes in their metrics match those associated with COVID-19. Anonymous data localized to regions such as neighborhoods or zip codes could provide public health officials and researchers a valuable tool to track and mitigate the spread of the virus, particularly during a second wave. Identifiable data, for example remote monitoring of cohorts (family, businesses, and facilities) associated with individuals diagnosed with COVID-19, can provide valuable data such as acceleration of transmission and symptom onset. This manuscript describes clinically relevant physiological metrics which can be measured from commercial devices today and highlights their role in tracking the health, stability, and recovery of COVID-19+ individuals and front-line workers. Our goal disseminating from this paper is to initiate a call to action among front-line workers and engineers toward developing digital health platforms for monitoring and managing this pandemic.

## Introduction

### Overview of COVID-19

The Coronavirus Disease 2019 (COVID-19), first recognized in December 2019 in Wuhan, China, is the latest respiratory disease pandemic currently plaguing global health. It has been shown to be caused by a novel coronavirus, severe acute respiratory syndrome coronavirus-2 (SARS-CoV-2), that is structurally related to the virus that causes SARS. Li et al. defined a suspected COVID-19 case as pneumonia that matched the following four criteria: (1) fever, with or without a recorded temperature; (2) radiographic evidence of pneumonia; (3) low or normal white-cell count or low lymphocyte count; and (4) no reduction in symptoms after antimicrobial treatment for 3 days (1). As its name suggests, the leading cause of fatality from COVID-19 is hypoxic respiratory failure (2–4). COVID-19 has posed significant challenges for the medical and civilian communities analogous to what was experienced in two preceding instances of the SARS-CoV virus outbreak in 2002 and 2003 and the Middle East Respiratory Syndrome (MERS) in 2012 (1, 5, 6). Importantly, Li et al. studied 425 patients with confirmed COVID-19 in Wuhan and estimated that the basic reproduction number (*R*_0_) for SARS-CoV-2, at the time, to be 2.2 (1). This suggests that each infected person, on average, can spread the infection to an average of 2.2 other people. The virus will likely continue to spread unless this number falls below 1.0 (5). Moreover, timely and effective containment strategies have been a cornerstone of managing the COVID-19 outbreak and reducing viral transmission.

### Return to Daily Living Post-COVID-19: From Testing to Digital Health Implementation

Most plans for recovery and the return to “normal,” every-day life are centered on testing—namely determining those who currently have an infection and those who have developed antibodies against the virus, indicating a possible recovery. With any test, there may be false positive or false negative results (7). Of note, an antibody test, while useful in quantifying the number of cases that have occurred in a population, is typically not suitable for *early* disease detection and its association with immunity to the virus has been put into question (8). Additionally, there is considerable cross-reactivity between SARS-CoV-2 and four other coronaviruses, including those associated with the common cold (9). Polymerase chain reaction (PCR)-based tests are highly sensitive and specific in the laboratory setting; however, high costs and limited availability make these tests difficult to suit population health needs. In the face of a pandemic, time is of the essence and researchers must think of new ways to improve disease diagnosis and monitoring of disease progression.

**Figure 1:**
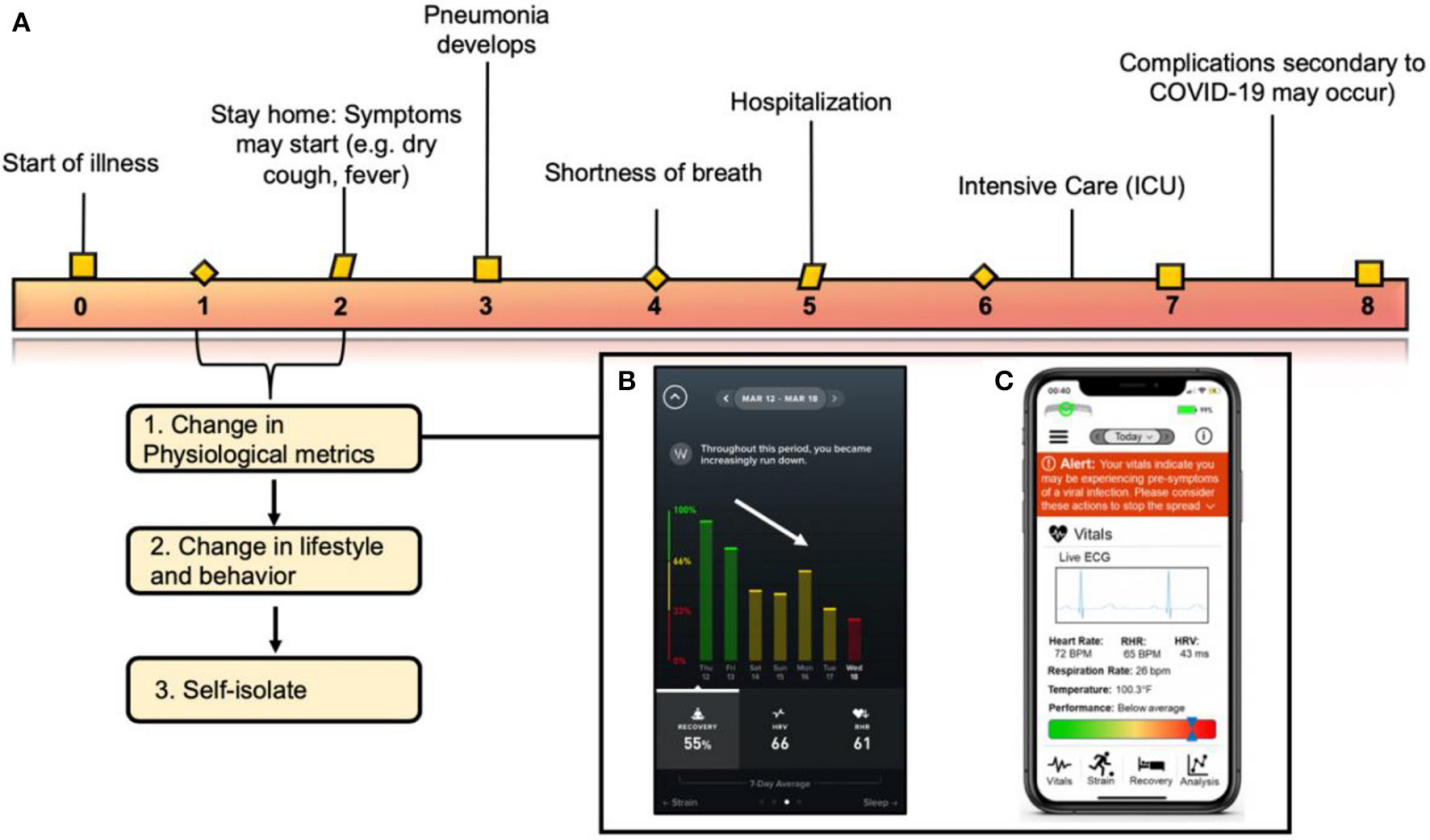
Correlating clinical symptoms to the immune response of COVID-19 and when the implementation of wearable technology fits into the virus timeline. **(A)** Correlating symptoms to the immunological pathway of COVID-19 described in this figure. Wearable sensors can be used to detect changes in physiological metrics before a formal diagnosis. **(B)** Image from the WHOOP application correlating a decrease in recovery with a diagnosis by an individual diagnosed with COVID-19 on Wednesday the 18th (red bar). **(C)** Schematic detailing an example of an iPhone application collecting physiological data from a wearable sensor and translating those metrics to alert an individual on his/her overall health status. Figure reproduced and modified from Azuravesta Design (10), **(A)** and Team (11), **(B)**.

With new tests in clinical trials, we believe there is an opportunity to leverage advances in remote patient monitoring technology to assist in early disease detection and monitoring by analyzing systemic infection precursors (Figure 1). Wearable sensor data may enable providers and patients to be alerted of a potential SARS-CoV-2 infection before symptoms become severe (Figure 1A). Importantly, a recent study showed that individuals with pre-existing hypertension, heart disease, or diabetes, which makes up nearly half of the United States population, had higher rates of intensive care hospitalization and death when diagnosed with COVID-19 (12). Additionally, data suggests that this vulnerable patient population also typically underreport their symptoms (13–15), making remote detection of disease through objective measures a possible way to improve timely escalation of care. On a larger scale, hospitals could use localized, de-identified data to track the spread and severity of the outbreak without violation of users' privacy to provide population-level care (Figures 1B,C). This becomes more relevant when one considers that the asymptomatic carrier rate is estimated to be between 25 and 50% of the entire United States population (16, 17). With such a large population potentially carrying the virus, digital health technologies that measure physiologic parameters can be leveraged to help identify population clusters to identify an emerging COVID-19 outbreak. Harnessing this information is feasible as ~16% of the United States population (~52.8 million people) currently have a smartwatch (18). Such technology may enable a more precise approach for subsequent more advanced testing (e.g., physiological testing), contact tracing, and quarantining. To further incentivize the adoption of such technologies, we envision companies that produce wearables will continue to work with insurance providers and other governing bodies to make these devices more accessible to the public (13). Most recently, in Germany, the Robert Koch Institute (equivalent of the United States Center for Disease Control and Prevention, CDC) supported the adoption of a smartphone app (Corona-Datenspende) which tracked temperature, pulse, and sleep from a minimum of 10,000 volunteers wearing smartwatches or fitness trackers with the aim of understanding how much of the population is clinically symptomatic from an influenza-like illness (ILI) (14). To date, more than 160,000 people have already enrolled (15). Results from the app will be displayed on an interactive online map, enabling both health authorities and the general public to better assess the prevalence and community distributions of infections (14). In the United States, a study published in early 2020 from the Scripps Research Institute demonstrated the ability to predict “hot spots” for influenza utilizing resting heart rate and sleep data from a smartwatch or fitness band (16). The team analyzed data from more than 47,000 consistent Fitbit users in five states (California, Texas, New York, Illinois, and Pennsylvania) over a 2-years period and found that when a cluster of individuals in one-region presented with increased heart rate, a subsequent rise in ILIs was detected. These models to map the prevalence of ILIs have correlated well with CDC data in the range of 0.84–0.9 (16). These studies highlight the clinical applications of wearable sensor technology and in the case of a pandemic, where “flattening the curve” is critical to limiting disease morbidity and mortality, such tools have the potential to improve health at the population level.

## Measurement of Physiological Metrics From Wearable Sensors for COVID-19 Monitoring

COVID-19, along with other viral illnesses, is associated with several physiological changes that can be monitored using wearable sensors (Table 1). Many metrics derived from heart rhythm such as heart rate (HR), heart rate variability (HRV), resting heart rate (RHR), and respiration rate (RR) could serve as potential markers of COVID-19 infection and are already measured by wearable devices such as the Apple Watch, WHOOP Strap, Fitbit, Zephyr BioHarness, or VivaLNK Vital Scout (Table 2). Additionally, changes in electrocardiogram (ECG) waveforms could contain information indicative of an infection. Many wearables report more complex metrics such as stress, recovery, activity, and sleep, which are typically calculated using a combination of cardiac and accelerometer-derived metrics. Due to the integration of multiple measurements, these metrics should exhibit an aggregate higher signal to noise ratio (SNR) than individual raw signals alone and, therefore, have higher predictive value. Core body temperature and arterial oxygen saturation (SpO_2_) are also of clinical value due to the high prevalence of fever and respiratory symptoms in COVID-19; however, such measurements are not routinely measured by the commercial wearables today. Furthermore, as patient-centered quality metrics are considered, we hypothesize that wearable devices, once validated via rigorous longitudinal randomized controlled trials, can decrease invasive metrics derived from arterial blood gas procedures (intended to detect how well lungs move oxygen into the blood) or from obtaining cardiac troponins (indicative of myocardial injury) (17).

**Table 1:** Sensor modalities for monitoring physiological metrics relevant to COVID-19.

	**HR**	**Heart rhythm**	**HRV**	**RHR**	**RR**	**SpO_**2**_**	**Skin temp**	Core temp^*^	**Sleep**
ECG	x	x	x	x	x			x	
PPG	x	x	x	x	x	x		x	
Accelerometer					x				x
Temperature							x	x	

*HR, Heart Rate; HRV, Heart rate variability; RHR, resting heart rate; RR, respiration rate, SpO_2_, Blood Oxygen Saturation; ECG, Electrocardiogram; PPG, photoplethysmography; Temp, Temperature*.

* *Core Temperature is measured based on HR and skin temperature and cannot be measured as a stand-alone metric*.

**Table 2:** Sampling of commercial wearable sensors used to monitor physiological parameters necessary for COVID-19 detection.

**Company and device**	**Form factor**	**CT**	**ST**	**SpO_**2**_**	**RR**	**HR**	**HRV**	**EDA**	**Other**	**Price ($)**	**FDA**
AIO Sleeve 2.0	Arm sleeve			y		y	y		Act, ECG	$169	n
Apple Watch Series 4/5	Wrist monitor				y	y	y		Act, ECG	$399	y
Beddit	Contactless In-bed sensor				y	y			Slp	$150	n
Beurer SE80	Contactless In-bed sensor				y	y			Slp	$500	n
Biobeat	Wrist monitor, Chest Patch		y	y	y	y	y		BP, ECG	NA	y
BioIntellisense	Epidermal patch		y		y	y			Coughing, sneezing, freq	NA	y
Biostrap	Wrist monitor			y	y	y	y		Slp	$175-320	n
Biovotion Everion	Armband		y	y	y	y	y		Slp	NA	n
Cosinuss Two	In-ear	y		y		y	y		Act	$330	n
Empatica Embrace	Wrist monitor		y			y	y		Act, EDA	NA	y
Equivital LifeMonitor	Chest belt	y	y	y	y	y	y		GSR	NA	y
Fitbit Charge 4	Wrist monitor			y		y			Act, Slp	$150	n
Fitbit Ionic	Wrist monitor			y		y			Act, Slp	$250	n
Fitbit Versa 2	Wrist monitor			y		y			Act, Slp	$200	n
Garmin Fenix 5	Wrist monitor			y		y			Act, Slp	$500	n
Garmin Forerunner 945	Wrist monitor			y	y	y			Act, Slp	$550	n
Garmin Venu	Wrist monitor			y	y	y			Act, Slp	$300	n
Garmin Vivoactive 4	Wrist monitor			y	y	y			Act, Slp	$270	n
Hexoskin	Compression shirt			y	y	y	y		Act, Slp	$579	n
Kinsa	Smart thermometer		y							$50	n
Oura	Ring		y		y	y	y		Act, Slp	$299	n
Spire Health Tag	Tag attached to clothing				y	y			Act, Slp	$399	n
VivaLNK Fever Scout	Epidermal patch		y							$60	y
VivaLNK Vital Scout	Epidermal patch				y	y	y		Act	$150	y
WHOOP	Wrist monitor				y	y	y	y	Recovery, Slp	$30	n

*Act, activity; BP, blood pressure; CT, core temperature; EDA, Early Detection Algorithm for viral illness or wellness prediction; ECG, electrocardiogram; EDA, electrodermal activity; Freq, Frequency; GSR, galvanic skin response; HR, heart rate; HRV, heart rate variability; NA, price not available online; RR, respiratory rate; Slp, sleep measures; SpO_2_, oxygen saturation; ST, skin temperature. Table, Data used for table gathered from news reports, social media sites, and from Google Docs (19)*.

The upcoming sub-sections in this paper will focus on the current role wearable sensors in providing remote patient monitoring for COVID-19. Our goal in each of these sub-sections is to (1) summarize the clinical relevance of each physiological metric as it relates to COVID-19, (2) provide a brief technical overview of each parameter detection modality, and (3) provide a brief overview of patient implications as it relates to quality of care. Discussion of current clinical trials utilizing commercially available, off-the-shelf (COTS) wearable devices pertinent sensors to COVID-19 is included to highlight the current work in this domain (Table 3).

**Table 3:** Current clinical trials utilizing commercial wearable sensor devices to diagnose and monitor COVID-19.

**Study name**	**Institution(s)/companies**	**Data source(s)**	**Focus of study**	**Clinical trials registry/ref**
N/A	Central Queensland Univ; Cleveland Clinic	WHOOP Strap 3.0	Correlating changes in respiration rate to predicting COVID-19	N/A
COVIDENTIFY	Duke	AWs, Fitbits, Garmin	Predicting and assessing severity of contracting Covid-19 or influenza from wearable sensors and wellness surveys	N/A
DETECT study	Scripps Research Institute Stanford Univ	Fitbit, Apple Watch, Garmin, Amazefit, OURA, Beddit, etc	Determining whether changes in heart rate, activity, sleep, or other metrics might be an early indicator for COVID-19 or other viral infections	NCT04336020
COVID-19 detection study	Stanford Univ	Fitbit, Garmin, Apple Watch, and Oura	Enrolling subjects who are at higher risk of exposure.	N/A
TeamPredict	University California San Francisco	Oura Ring	Correlating changes in skin temperature and heart rate to COVID-19	N/A
Kinsa	N/A	Smart Thermometer	Correlating changes in skin temperature and social distancing guidelines to COVID-19	N/A

*Table is put together based on press releases found on social media platforms and in the news. Aws, Apple Watch; Univ, University; Ref, References; N/A, Not applicable*.

### Cardiovascular Monitoring

There are several metrics related to cardiac function such as HR, HRV, and heart rhythm wherein changes in these metrics may be indicative of COVID-19 infection. Viral illness increases physiological stress on the body which typically manifests as an overall increase in HR. In many cases of viral infection, an elevated HR can be detected hours or days before the onset of symptoms (20). Elevation in HR is also a typical physiological response during fever as the body begins to mount a defense to infection (21). An increase in RHR can be indicative of systemic illness, and thus RHR data, on a population scale, has been proven to accurately model the outbreak of influenza (as previously described) (16). HRV, measured as the average time difference between heart beats, provides insight into overall health, performance, and stress of an individual. High HRV is associated with fitness and health (22). A significant decrease in HRV indicates inadequate recovery and is indicative of increased physiological stress (23). While there is a lack of clinical evidence on the predictive value of HRV for viral illness detection, there is a large amount of self-reported and anecdotal evidence which leads us to postulate that HRV trends can be used to predict the onset of illness (23). Researchers at Scripps recently launched the Digital Engagement and Tracking for Early Control and Treatment (DETECT) study which seeks to correlate changes in HR to the incidence of acquiring a viral infection such as COVID-19 (24, 25). While other viral illnesses are being studied as well, the primary objective of this study is to assess HR, activity, and sleep data in 100,000 individuals to identify ILIs via the CareEvolution's myDataHelps application (24). The study, which commenced this past March, will utilize the Apple Watch, Garmin watch, and Fitbit, which are connected to Apple Health, Amazefit, or Google Fit platforms, respectively. Another study by the team at Scripps Research Institute, in collaboration with Stanford University and Fitbit, is assessing whether changes in HR, skin temperature, and SpO_2_ can be used to predict the onset of COVID-19 before symptoms even start (26). These studies build upon the work published earlier this year by Scripps in correlating changes in HR to influenza (16).

Electrocardiogram (ECG) and photoplethysmography (PPG) are widely used in wearable technology to monitor cardiac function (27–30). ECG is a measurement of the electrical activity in the heart, and PPG uses light (at specific nanometer wavelengths) to measure changes in blood volume (27, 31). While ECG sensors are typically implemented in the form of an epidermal patch that adheres to the stratum corneum (e.g., Zio Patch) and/or via leads to a benchtop instrument, the commoditization of wrist-worn monitors with predictive algorithms has enabled the measurement of heart rhythms from wearable devices such as the Apple Watch 4 and 5, although this measurement is not continuous (32, 33). On the other hand, PPG can be measured continuously in many locations on the body including the wrist, fingertips, earlobes, torso, and more (31). In this sense, PPG is more versatile and can be implemented in more form factors including watches and earbuds (27). While both are viable to monitor the metrics discussed above, ECG is a more direct measurement of heart activity which could potentially provide more insight toward the onset of COVID-19. There is growing evidence suggesting that COVID-19 is burdened by a higher risk of arrhythmic events (34). A study by Driggin et al. found that in 138 hospitalized COVID-19 patients, arrhythmias such as ventricular tachycardia/fibrillation represented the leading complication (19.6%) after acute respiratory distress syndrome, particularly in those admitted to intensive care unit where the prevalence rose to 44.4% (35). Future work toward moving this field forward, leveraging data analytics and wearable sensors, could involve detecting such arrhythmias in patients with COVID-19 in a real-time manner toward improving patient outcomes.

### Cardiovascular Strain, Sleep, and Activity Levels

Many currently available wearable devices provide users with calculations of advanced metrics such as stress or strain, sleep, activity, and recovery. These metrics typically rely on a combination of measurements and are calculated daily. The combined measurements and long measurement time for these metrics should yield a higher SNR and thus will likely be better predictors of COVID-19 infection than any single raw metric. Cardiovascular stress or strain (the terms are used interchangeably between analytical platforms) is expressed as a dimensionless unit derived from a combination of HR and HRV data measured over a given day. For the purposes of this paper, we will use the word strain. Devices such as WHOOP measure cardiovascular strain based on time spent in HR zones. In the context of athletic performance, a field where cardiovascular strain has been extensively studied to modulate the internal workloads of athletes (27–30, 36), an individual undergoing a strength-based workout with minimal reps and periods of rest will have a lower strain if their HR is not elevated for extended periods of time (27, 36, 37). Increasing weight and adding new strength exercises will cause muscle soreness and muscle fatigue. This microtrauma from the eccentric lengthening of the muscle fibers will cause a decrease in HRV especially in the morning. Fatigued muscles will result in higher strain as the day progresses because the body is working harder to recover due to the disturbances in the individuals' homeostatic state. Along the same lines, cardiovascular strain is also expected to increase when fighting a viral infection. A viral infection such as influenza or COVID-19 does this by increasing the stress on the cardiovascular system, indicated by increases in RHR, HR, blood pressure, and an intrinsic stress hormone called catecholamines (38). Sleep is usually detected using a combination of HR patterns and accelerometer data. Sleep quality is assessed primarily through the analysis of HR, RHR, and HRV, but accelerometry may be used to determine disturbances. Elevated sleep duration has been shown to be predictive of ILI. An increase in sleep duration paired with a decrease in sleep quality would be expected to occur in COVID-19 cases. Activity metrics are intended to report the amount of physical exertion for a day or a given timeframe. Activity scores are typically based on periods of elevated HR and accelerometry. While changes in activity data may not be particularly useful for individual treatment or diagnosis, general trends in activity data for a large population could likely be used for pandemic modeling or to study the health effects of social distancing and isolation. Martin et al. studied the relationship between exercise and respiratory track viral infections in small animal models and concluded that moderate intensity exercise reduced inflammation and improved the immune response to respiratory viral infections (39). The use of wearable sensors toward monitoring activity levels could provide an objective means of staying physically active and healthy during the COVID-19 pandemic. Recovery assessments are based on sleep, sleep quality, and HRV. There is a growing amount of evidence showing a clear downward trend in recovery scores in the days leading up to the onset of COVID-19 symptoms. These complex metrics may prove to be reliable indicators of COVID-19, but it is important to consider that each wearable device uses different algorithms and measurements for the calculation of these scores, which severely limits the population on which analysis of these metrics can be performed.

### Respiration Monitoring

Respiration rate (RR) is of critical interest in COVID-19 cases due to the severe effects the virus can have on the lungs. COVID-19 presents as a lower-respiratory tract infection in most cases which can cause inflammation of lung tissue, coughing, and shortness of breath (40). The respiratory damage caused by COVID-19 reduces the overall efficiency of the lungs which results in an increased RR to compensate (40). Significantly increased RR is not as common in cases of other viral illnesses such as influenza or the common cold because these viruses typically affect the upper-respiratory tract (40). It may be concerning, however, that by time the patient is tachypneic, the disease may already be in an advanced stage. In a person who has a high likelihood of COVID-19 exposure, a device that is able to detect subtle changes in respiratory function prior to the onset of clinical symptoms, such as shallow respirations, wheezing, and shortness of breath, has the potential to be an effective tool. Of note, findings by Luo et al. indicated that as many as 70% of frontline health care workers are testing positive for COVID-19 (41). Frontline staff who care for patients with COVID-19 could benefit from the remote use of a wearable-sensor remote monitoring mechanism to objectively monitor for pre-clinical signs of infection, as a measure to prevent spread to other colleagues or patients. Additionally, current COVID-19 guidelines suggest that measuring resting RR can be used utilized as a criterion for intensive care unit (ICU) admission (42).

A recent review by Massaroni et al. assessed the suitability of different contact-based techniques for monitoring RR in clinical settings, occupational settings, and sports performance (43). Specifically, in the context of clinical settings, the authors noted that contact-based techniques such as strain, impedance, biopotential, and light intensity measurements offer a platform to detect RR in a non-invasive and unobtrusive manner. Toward the use of biopotential measurements for RR monitoring, RR can be derived from wearable devices that measure heart activity due to a phenomenon known as Respiratory Sinus Arrhythmia (RSA) (44). RSA results in increased HR during inspiration and decreased HR during expiration. Using this information, any wearable that can accurately measure heart rhythm can be used to derive respiration rate if an appropriate algorithm is implemented. Baseline resting of respiration rate can be determined when a subject is asleep and shows very little variation from night to night (40). Therefore, a significant increase in resting respiration rate indicates a high likelihood of decreased respiratory efficiency. WHOOP has focused on correlating changes in RR and recovery levels to predicting COVID-19 in their users (45). WHOOP, which recently partnered with Central Queensland University (CQU) and the Cleveland Clinic on such a study, will utilize the data collected from WHOOP's hardware from volunteers who have self-identified as having contracted COVID-19 to study changes in their respiration rate over time (45). The data, which is currently being collected for this study utilized the WHOOP 3.0 strap, was validated externally to determine the accuracy of respiration rate during sleep when compared against polysomnography (46). Based on the study, the team from WHOOP hypothesizes that measuring respiration rate during sleep could be valuable in detecting abnormal respiratory behavior in COVID-19 patients before symptoms are present (45). Recently, researchers from Duke University launched the “CovIdentify” study which utilizes devices such as the Fitbit and data from the Apple Health app to monitor an individual's sleep schedules, oxygen levels, activity levels, and HR over a 12 months period to determine if they are risk for COVID-19 (47, 48). Once the data is collected, the team will utilize their predictive algorithms to detect respiratory infections from the COVID-19 virus. Respiration rates are typically obtained in research and clinical-related settings which may not be indicative of individual's respirations at home; however, given that COVID-19 can complicate existing chronic respiratory disease, monitoring individuals in home settings can receive a more patient-centered approach to prescribing treatment (49). There remains an unmet medical need to ensure that algorithms that correlate changes in RR to COVID-19 are sensitive enough to filter out other lower respiratory infections such as pneumonia or influenza. Toward achieving this goal, the design of clinical trials to mitigate false positive diagnosis is critical toward the application of wearable sensor technology for COVID-19 monitoring.

### SpO_2_

The assessment of a patient with a respiratory illness typically includes measurement of the blood oxygen saturation (SpO_2_), as hypoxia in certain clinical scenarios is indicative of a pneumonia. This is of particular importance in monitoring progression and severity of disease in COVID-19, where resting SpO_2_ was found to be significantly lower in patients with a severe stage of the disease as determined by clinical symptoms and CT scan. SpO_2_ measurements < 90% during hospital admission is seen in COVID-19 patients with higher systemic inflammatory markers and increased disease mortality (50, 51). While validated oximeters are abundant in the inpatient setting, few patients have this technology available in their homes. Smartphone-based pulse oximetry in the form of a camera-based app and a probe-based app, the latter using an external plug-in probe, have been developed by several companies and evaluated in two published studies (52, 53). The plug-in probes showed modest accuracy in identifying hypoxia as measured by standard pulse-oximetry; however, the camera-based technology had limited ability to accurately detect hypoxia and is not considered standard of care for this purpose in the literature. PPG technology has been utilized in wearable pulse oximetry and a large number of fingertip-type oximeters are commercially available, but few meet acceptable accuracy standards (54). Examples include the MightySat™ Rx (Masimo) and Pulsox-310 (Konica Minolta). These available technologies were designed for oxygen management in chronic diseases such as chronic obstructive pulmonary disease (COPD) and sleep apnea; moreover, little research has looked at their utility in early detection and management of disease progression in acute respiratory illnesses. At present, this technology may be particularly useful, as more patients with mild symptoms of COVID-19 are being asked to stay at home and report changes in their respiratory symptoms via telemedicine modalities in an attempt to reduce the spread of the disease. In elderly patients or those with medical co-morbidities that are known to be at higher risk of disease progression, current wearable PPG technology may have a role in identifying those patients who are self-isolating at home that need a higher level of care due to hypoxemia which may or may not be accompanied by other clinical symptoms of early respiratory distress.

### Temperature

Temperature measurement is extremely important to COVID-19 detection and has already been widely used by numerous countries as an immediate test to determine if travelers or citizens may be infected with COVID-19. While quarantining individuals with fever may prevent transmission to some degree, this approach to temperature monitoring is not sufficient because COVID-19 can be transmitted before a fever develops. Continuous monitoring of skin temperature is currently being implemented using wearable devices such as the TempTraq, Oura ring, VivaLNK Fever Scout, and QardioCORE. The TempTraq skin temperature sensor adheres to the body for 72 h and is currently being used to measure the temperature of frontline workers here at University Hospitals Cleveland Medical Center (55). A study performed by Stanford University using the MOVES, Scanadu Scout, Basis B1, Basis Peak, iHealth-finger, Masimo, RadTarge, and Withings found a notable increase in skin temperature as well as HR and RHR in the period preceding and during a viral infection (20). This change in skin temperature, particularly if paired with RHR and HRV information, could be used to predict COVID-19 infection before symptoms arise. Any such temperature sensing wearable device could also be used for fever tracking during illness and alert users and medical staff to a dangerous fever or sharp change in temperature. While skin temperature measurement is easy to implement, it has been shown to deviate up to 12°F from core body temperature (20). Additionally, temporal, oral, aural, and axillary temperature measurements have all been shown to be invalid estimations of core body temperature (compared against rectal thermometry) and are more prone to change due to environmental or behavioral factors (56). Core body temperature measurement provides a much more stable baseline for assessment and could prove to be more reliably indicative of illness than skin temperature and provide more insight into fevers for remote patient monitoring. Researchers at UT Southwestern Medical Center found that fluctuations in core body temperature regulate the body's circadian rhythm (57). In the study, the researchers focused on cultured mouse cells and tissues and found that genes related to circadian functions were influenced by changes in core temperature. Clinically, analytical platforms combining core body temperature measurements with those of respiration rate, HR, or HRV, could provide a more robust platform for predicting the incidence of COVID-19 in ways not done today.

Continuous skin temperature measurement is simple to implement in both hardware and software and can easily be implemented into a wearable device. Analog solutions such as thermocouples and thermistors could be used reliably, but digital temperature sensors are likely better for wearable applications due to their small size (~1 × 1 mm), low power requirements, and improved control. Such a sensor could be integrated into many existing wearable form factors though adhesive patches will likely prove more reliable due to constant contact with the skin. The gold standard for core body temperature is rectal thermometry. This measurement modality is not feasible for continuous measurement where non-invasive and unobtrusive monitoring is required. A large body of research has shown that core body temperature can be reliably predicted from skin temperature and HR through the use of Kalman filters or other machine learning (ML) algorithms (58, 59). While this technology needs further research and development before clinical or diagnostic use, the application of such algorithms could provide a non-invasive method to study the response of core body temperature to illness and provide advanced remote patient monitoring capabilities for fever treatment.

While there have not been any clinical trials correlating core body temperature to incidences of COVID-19, the smart thermometer maker, Kinsa, has shown from skin temperature data where people with the flu (and more recently COVID-19 infections) are located (60). The team studied a population in Miami-Dade County, Florida and found that a spike in fevers coincided with the well-known reports of Miami residents and tourists loosely following social distancing recommendations. As beaches closed and other isolation strategies were implemented in the county, the team found a significant drop in fevers. The team also noted that the trends observed in Miami hold true for other areas of the country that they studied: as individuals adhered to social distancing guidelines, within 5 days, a downward dip in fevers was observed (60). Another start-up, Oura, has partnered with the University of California, San Francisco on a new study to see if its device, Oura Ring, can detect physiological signs that may indicate the onset of COVID-19 (61). The study includes two parts wherein part one involves having 2,000 frontline healthcare professionals wear the Oura Ring to track skin temperature, sleep pattern, HR, and activity levels. Part two of the study will involve Oura's general user population wherein its 150,000 global users can opt in to participate and add to the overall pool of information with their ring's readings and daily symptom surveys (61). Recently an Oura user in Finland claimed that the ring alerted him that he was displaying symptoms of COVID-19 based on decreased recovery levels (from 80 to 90% to 54 coupled) and an increase in skin temperature of ~1°C. These changes prompted the individual to get tested (61). The test results confirmed, that while asymptomatic, the individual had COVID-19 (61). The compilation of de-identified data sets from studies such as the two mentioned for temperature monitoring and those mentioned earlier on could lead to the development of an early detection algorithm.

## Early Detection Algorithm Technology is Needed for COVID-19 Monitoring

**Figure 2:**
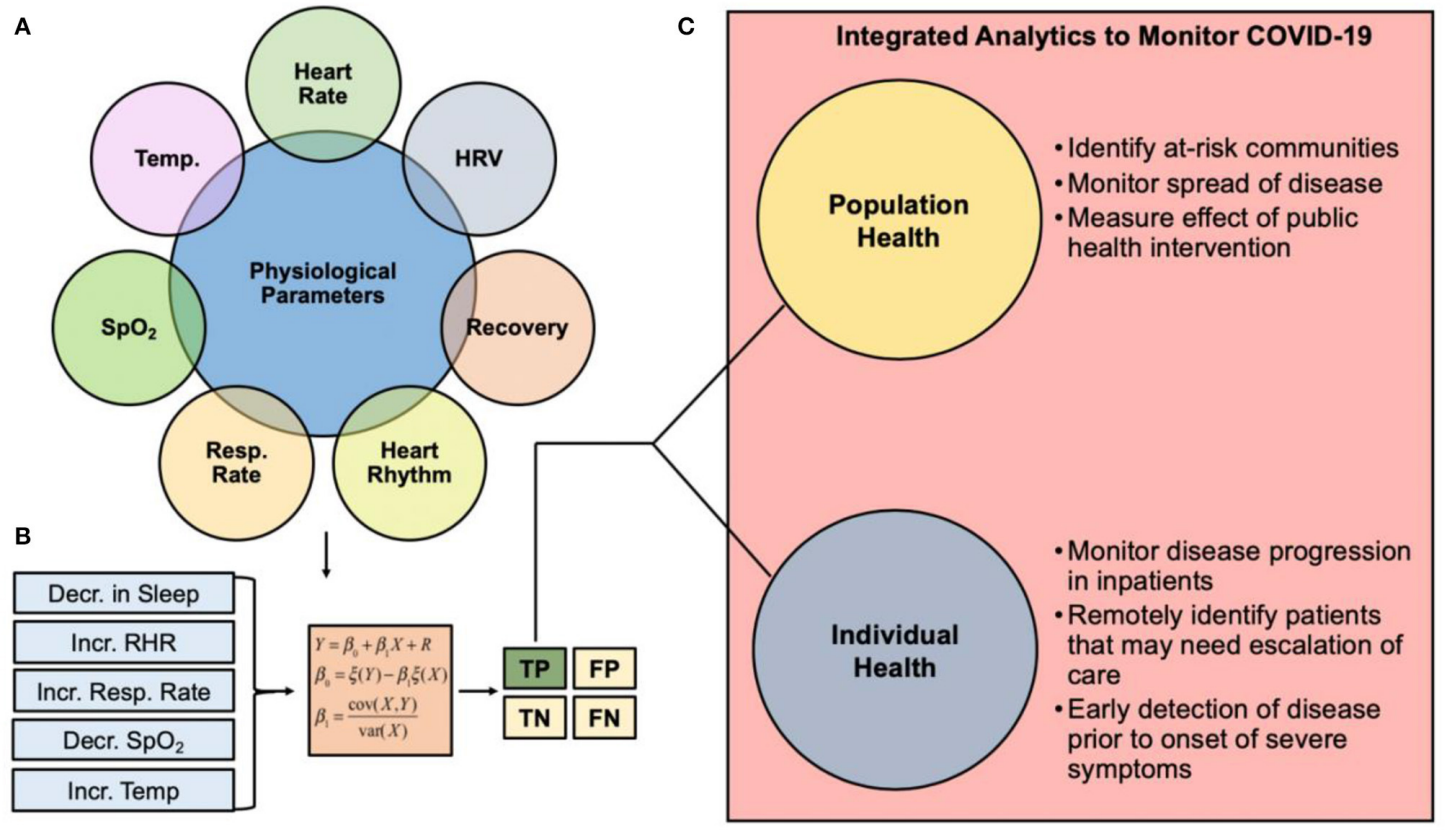
Clinical pathway summarizing the role of wearable sensor technology and predictive analytics for monitoring COVID-19. **(A)** Physiological metrics currently capable of being measured from commercial wearable sensors. **(B)** Changes in physiological metrics can be inputted into an early detection algorithm for COVID-19 monitoring. The goal of such algorithms is to ensure the true positive rate is robust to support the use of the analytics for real-time clinical decision making. **(C)** Integrated analytics to monitor COVID-19 can be used to monitor individual or population health. HRV, heart rate variability; Resp Rate, respiration rate; SpO_2_, blood oxygen saturation; Temp, temperature; TP, True Positive; FP, False Positive; TN, True Negative; FN, False Negative.

Many of the physiological changes measured by wearable devices discussed in the above sections can potentially be detected before a user experiences any significant clinical symptoms of illness (Figure 2A). We postulate that wearable devices can detect and alert users of possible infection with SARS-CoV-2 before they develop clinical symptoms through the development of an early detection algorithm (EDA) (Figures 2A,B). By notifying wearable device users of possible early infection, EDA could allow them the ability to self-isolate, seek care or diagnostic testing, and take other steps to mitigate transmission of the infection during a critical period of the disease process. Additionally, wearables could be used for remote patient monitoring in mild cases by allowing patients to report their vitals from home, saving critical hospital resources and reducing the risk of transmission to health care providers by avoiding in-person assessments (Figure 2C). A combination of the metrics listed above could result in a sufficiently high SNR to be used as a predictor of viral illness or COVID-19 risk. Developing an EDA with a high true positive and true negative rate is imperative for the translation of this technological platform for remote patient monitoring. Clinical staff such as intensive care nurses use early-warning system indicators to detect if individuals are at risk for further complications related to their care (62). Remote patient monitoring using wearable sensor technology provides an opportunity for developing more effective patient interventions, balancing nurse-patient care ratios, and decreasing costs associated with readmission rates and futile medical care.

## Future Outlook and Recommendations: Adopting Wearable Sensor Technology

The development of integrated sensor technology has made it possible to remotely measure many physiologic parameters accurately, many of which are clinically useful in monitoring disease progression in a viral illness. The scope of influence of this technology is broad; it may be used to help to identify an individual under home-quarantine that needs a higher level-of-care or a community where an emerging outbreak may be imminent and requires an early intervention. We suspect that one of the largest impediments for the mass adoption of wearable sensors (and digital health technologies overall) in the United States toward remote patient monitoring is the issue of data privacy, data sharing, and underreporting. Wearable technology companies must ensure that only users who choose to participate will share their data (as done by WHOOP), and that the data will be anonymized and used for COVID-19 research only. Germany have provided us with a good example of how population health data can be handled, acknowledging their strong privacy concerns and stances on the limited collection of digital data (63). Underreporting of data by some populations may require their consent for safe data sharing and privacy agreements so that it can be used to inform better care, thus decreasing health disparities. The implementation of current trials demonstrates the convergence of wearable data, self-reported symptoms, molecular testing, and geospatial data toward developing platforms for managing COVID-19 and other outbreaks which may arise in the future. Building upon such trials, we see an opportunity to design a device that can accurately monitor many or all metrics of interest and through machine learning is able to develop an algorithm to reliably detect changes in population health status. A collaboration leveraging the expertise of clinicians, data scientists, engineers, and nurses is imperative to facilitate this advancement and may even be more acutely desired should there be a second wave of this pandemic.

## Author Contributions

DS, ED, EH, and SK wrote and edited the manuscript. TW, JH, JV, and CD contributed to the editing of the manuscript. All authors contributed to the article and approved the submitted version.

## Conflict of Interest

The authors declare that the research was conducted in the absence of any commercial or financial relationships that could be construed as a potential conflict of interest.
